# Psychological Resilience: An Affect-Regulation Framework

**DOI:** 10.1146/annurev-psych-020122-041854

**Published:** 2022-09-14

**Authors:** Allison S. Troy, Emily C. Willroth, Amanda J. Shallcross, Nicole R. Giuliani, James J. Gross, Iris B. Mauss

**Affiliations:** 1 Popular Comms Institute, Lancaster, Pennsylvania, USA; 2 Department of Psychology, Franklin & Marshall College, Lancaster, Pennsylvania, USA; 3 Department of Psychological & Brain Sciences, Washington University in St. Louis, St. Louis, Missouri, USA; 4 Department of Population Health, New York University School of Medicine, New York, NY, USA; 5 College of Education, University of Oregon, Eugene, Oregon, USA; 6 Department of Psychology, Stanford University, Stanford, California, USA; 7 Department of Psychology, University of California, Berkeley, California, USA

**Keywords:** resilience, adversity, stress and coping, emotion, emotion regulation, affect regulation, psychological health

## Abstract

Exposure to adversity (e.g., poverty, bereavement) is a robust predictor of disruptions in psychological functioning. However, people vary greatly in their responses to adversity; some experience severe long-term disruptions, others experience minimal disruptions or even improvements. We refer to the latter outcomes—faring better than expected given adversity—as psychological resilience. Understanding what processes explain resilience has critical theoretical and practical implications. Yet, psychology’s understanding of resilience is incomplete, for two reasons: (*a*) We lack conceptual clarity, and (*b*) two major approaches to resilience—the stress and coping approach and the emotion and emotion-regulation approach—have limitations and are relatively isolated from one another. To address these two obstacles, we first discuss conceptual questions about resilience. Next, we offer an integrative affect-regulation framework that capitalizes on complementary strengths of both approaches. This framework advances our understanding of resilience by integrating existing findings, highlighting gaps in knowledge, and guiding future research.

## INTRODUCTION

Exposure to adversity—circumstances such as poverty or life events such as job loss, serious injury, or bereavement—is a robust predictor of disruptions in psychological functioning. Importantly, however, people vary greatly in the extent to which exposure to adversity leads to such disruptions (Bonanno et al. 2015, Masten 2018, Rutter 2006): While some people experience severe long-term disruptions, others show few or no long-term disruptions, or even improvement. Take, for example, two people who lose a loved one. After initial shock and grief, one person might continue to feel strong distress, hopelessness, and depression lasting several years. In contrast, a second person might experience the same initial shock and grief in the weeks following the loss; yet, they might soon begin to recover and, within some months, experience sadness but no lasting depression, and even feel an increased sense of meaning and life satisfaction. We refer to the latter outcomes—faring better than would be expected in a given cultural context—as psychological resilience.

What psychological processes explain resilience? Answers to this question are of significant theoretical importance in that they advance our basic understanding of the vast range of responses that people exhibit to adversity. In addition, given that adversity is an unavoidable fact of human life that places staggering burdens on individuals and societies, answers to this question have enormous practical importance inasmuch as they point to targets for increasing resilience. In the past few decades, psychological research on the processes that explain resilience has come from two major sources: the stress and coping approach and the emotion and emotion-regulation approach (Compas et al. 2017, Gross 2015, Lazarus 2000), henceforth referred to as the coping approach and the emotion-regulation approach, respectively. Each approach has yielded important insights. However, our understanding of resilience has been limited by two key obstacles. First, research on resilience has been slowed by lack of conceptual clarity. Second, the coping and emotion-regulation approaches each have limitations, and there has been a relative lack of cross-talk between the two. We address each of these obstacles in turn.

First, to contribute to conceptual clarity, we review six core questions encountered in research on resilience and provide an overview of the conceptual space by considering various ways these questions could be answered. We then locate our own approach within this conceptual space. Second, to integrate and build on the coping and the emotion-regulation approaches, we adopt an affect-regulation framework. This framework capitalizes on the complementary strengths of the coping and emotion-regulation approaches, organizes past findings, identifies gaps in existing knowledge, and helps guide future research.

## CONCEPTUAL CONSIDERATIONS IN RESILIENCE

As noted by others, “the theoretical and research literature on resilience reflects little consensus about definitions, with substantial variations in operationalization and measurement of key constructs” (Luthar et al. 2000, p. 544; see also Bonanno et al. 2015, Hiebel et al. 2021, Kalisch et al. 2015). Increasing conceptual clarity helps to resolve disagreements, summarize and synthesize research findings, identify the best methods and measures for particular research questions, identify crucial gaps in understanding and important next steps for research, and develop theories to programmatically articulate and test new hypotheses. To further these goals, we begin by laying out the conceptual space of resilience.

### The Conceptual Space of Resilience

In Table 1, we delineate six key questions that researchers encounter when thinking about resilience, possible answers to these questions, and how we have answered these questions.

The first question focuses on the level of analysis for resilience: As indicated in the first row of Table 1, the level of analysis can be individuals, groups (e.g., a family), and communities (e.g., a neighborhood, city, or country). While psychological research on resilience has often focused on individuals, there is increasing attention to groups and communities (Bonanno et al. 2015, Hall & Zautra 2010, Masten & Motti-Stefanidi 2020). Although these levels interact with and influence one another (Masten & Motti-Stefanidi 2020), resilience at one level does not imply resilience at another level (cf. Bonanno et al. 2015). Thus, an increasing number of researchers have adopted a systems approach, which involves conceptualizing resilience as the dynamic interplay among individual, family, and community levels (Hall & Zautra 2010, Masten & Motti-Stefanidi 2020).

The second question concerns the role of adversity in resilience. As shown in the second row of Table 1, this question can be answered in a number of ways. In some cases, resilience is conceptualized as a relatively stable trait that is present and measurable even in the absence of adversity (Block & Kremen 1996). In other cases, resilience is thought to occur only in response to adversity; that is, some type of adversity must be experienced to observe resilience (Bonanno et al. 2015, Compas et al. 2017, Kalisch et al. 2015). When adversity is considered, it needs to be defined. This is all the more necessary given that there are deep differences in how this can be done. One of the most consequential distinctions is between defining adversity in objective terms (e.g., events such as poverty, disaster, bereavement; Holman & Silver 1998, Masten & Motti-Stefanidi 2020) versus subjective terms (e.g., individuals’ appraisals that an event was adverse; Epel et al. 2018, Lazarus et al. 1985). Additionally, adversity can vary along dimensions such as intensity, timing, duration, controllability, globality, threat, and deprivation (Epel et al. 2018, McLaughlin & Sheridan 2016, Troy et al. 2013) and across types of events (e.g., bereavement, trauma; Lucas 2007), and we have to be clear about these distinctions and whether they matter. In sum, what constitutes or predicts resilience in one context may not do the same in another.

As shown in the third row of Table 1, the third question focuses on the nature of resilience (see Hiebel et al. 2021). Three main conceptualizations have been offered. First, resilience can be considered as a factor, or a relatively stable trait, that exists both in the presence and in the absence of adversity. For example, trait resilience has been conceptualized as the stable predisposition to adapt to change (Block & Kremen 1996, Waugh et al. 2008). Second, resilience can be considered as a process or a set of processes, such as capacities or resources that are deployed in response to adversity and allow individuals to withstand ongoing demands and maintain functioning. As described by Schetter & Dolbier (2011), resilience processes can include social (e.g., social support) and psychological (e.g., cognitive and behavioral strategies, including coping and emotion regulation) aspects. Third, resilience can be considered as an outcome, such as maintained or high functioning following adversity (Kalisch et al. 2015, Masten 2018). In turn, functioning can be indicated in a variety of ways. At the level of the individual, indices of resilience include well-being; psychological health; levels of psychopathology; number of symptoms; presence of diagnoses; academic, occupational, or social achievements; or accomplishing developmental milestones. At the level of groups and communities, indices of resilience include social and economic infrastructure and resources relating to education and human development (Hall & Zautra 2010). Resilience established by one indicator (e.g., academic achievement) does not necessarily imply resilience established by another indicator (e.g., psychological health) (Infurna 2020, Luthar et al. 2000), and it is thus important to be clear about the indicator of functioning that is used.

**Figure 1:**
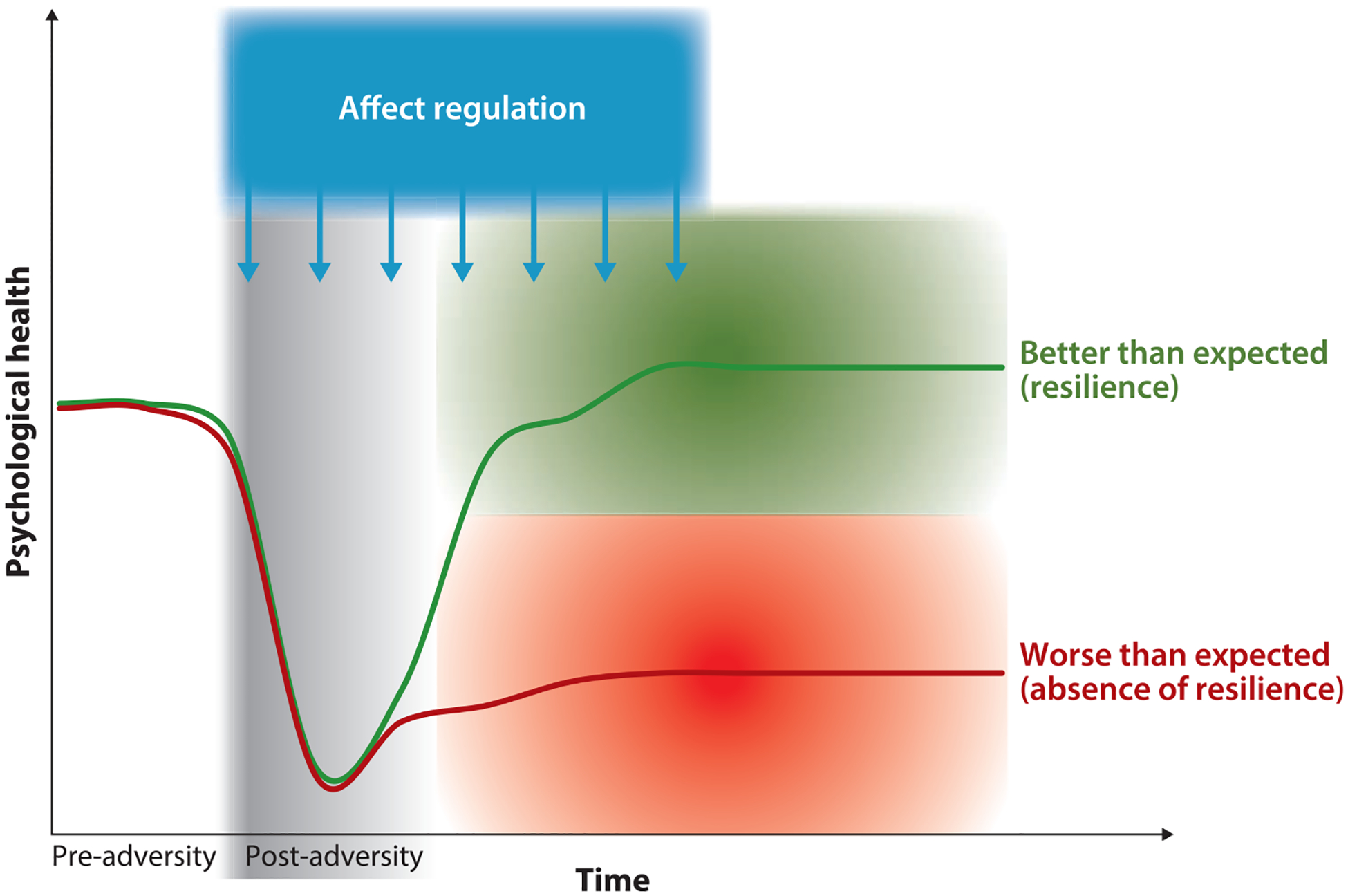
Our conceptual approach to psychological resilience. The two lines depict two prototypical trajectories: the green one leading to better-than-expected psychological health (resilience) and the red one leading to worse-than-expected psychological health (absence of resilience). The *x*-axis depicts time relative to adversity onset, indicating pre- and post-adversity. The *y*-axis depicts psychological health (i.e., resilience). The graded green and red backgrounds indicate that people fall along a continuum of resilience rather than into discrete types. The graded gray background indicates the gradual offset of adversity exposure.

Questions four through six (rows 4–6 of Table 1) concern the criteria by which resilience is established—that is, how we determine that an individual, group, or community is resilient. These three criteria-related questions can be understood in terms of a graph with a *y*-axis depicting functioning and an *x*-axis depicting time. We illustrate these questions in Figure 1 with regard to resilience outcomes, but they could be articulated for resilience factors or processes as well.

Question four (Table 1, row 4) asks what criterion for functioning is applied to determine resilience. In other words, at what point on the *y*-axis is an individual, group, or community considered resilient? A range of criteria could be used. First, one could set the criterion as meeting an absolute point such as scoring above or below a particular threshold (e.g., falling below the diagnostic criteria for posttraumatic stress disorder; Bonanno 2004, Waugh et al. 2008). Alternatively, resilience could be conceptualized in relative terms, that is, as high functioning relative to others who experienced similar adversity (Korol et al. 2002), relative to others who did not experience adversity (Jaffee et al. 2007), relative to oneself (e.g., returning to one’s pre-adversity levels of functioning; Infurna 2020, Lucas 2007), or relative to expectations given a particular cultural context and particular adversity (Luthar et al. 2000). In each case, one can conceptualize resilience as categorical (e.g., resilient versus not resilient) or continuous (for discussions, see Bonanno et al. 2015, Hiebel et al. 2021).

Questions five and six regard the *x*-axis, or, timing. As addressed in the fifth row of Table 1, first we need to ask what criterion for trajectory is applied to determine resilience. In other words, at what time point relative to adversity onset does resilience emerge? In some cases, such as in factor conceptualizations, resilience is thought to be observable at any point in time (Tugade & Fredrickson 2004, Waugh et al. 2008). If resilience is thought of as varying in time, one could decide that resilience must emerge during or immediately after encountering adversity (Bonanno et al. 2015, Kimhi et al. 2021). For example, to be resilient someone might have to exhibit greater functioning immediately after encountering adversity. At the other end of the continuum, one could presume that to be resilient, an individual, group, or community simply needs to show higher functioning at any point in their lives, which could be years or decades after experiencing adversity (Infurna 2020, Korol et al. 2002, Rutter 2006). For example, to be resilient someone might have to exhibit greater functioning 2 years after encountering adversity. As addressed in the sixth row of Table 1, we also need to ask what criterion for duration is applied to resilience. In other words, how long must indicators of resilience be present for someone to be considered resilient? There is a similarly wide range of possibilities here, ranging from a brief moment (Waugh et al. 2008) to years or even decades (Jaffee et al. 2007).

As Table 1 indicates, the key conceptual questions investigators encounter when thinking about resilience have been answered in a wide variety of ways (Kalisch et al. 2015). In our view, there is no single right answer; rather, one’s answers will depend on one’s research question and aims. However, it is crucial that researchers clearly state their position on each question (Luthar et al. 2000). We summarize our own position in the third column of Table 1.

### Our Conceptual Approach to Resilience

With regard to the first question (Table 1, row 1), we are interested in understanding how individuals adapt to adversity. Thus, our unit of analysis is the individual. However, we emphasize that all individuals are embedded within groups, communities, and cultural contexts that influence individual experiences and resilience, thus underscoring the importance of the interplay between different levels of analysis.

Next (Table 1, row 2), we conceptualize resilience as occurring in response to adversity. In turn, we define adversity as circumstances or events that many people in a given cultural context would expect to tax or exceed their resources and that have the potential to disrupt functioning. We focus on circumstances and events that are expected to disrupt functioning at least to a moderate extent and at least for a moderate amount of time (i.e., lasting a minimum of hours, days, or weeks). This definition of adversity (*a*) excludes events and circumstances that are very unlikely to affect functioning (e.g., a one-time, transient, mild event); (*b*) includes a wide range of circumstances and events (e.g., poverty, job loss, divorce, terrorist attacks, or global events such as the COVID-19 pandemic), including those that might not be considered adverse for all people and in all cultural contexts (e.g., a wedding, a move); (*c*) avoids concerns about circularity (i.e., adversity is what adversely affects a person) by using a consensus criterion rather than an individual-based subjective criterion; and (*d*) avoids the difficulty of establishing an entirely objective criterion by using people’s appraisals instead (i.e., what most people expect to potentially disrupt functioning). Thus, our definition of adversity is broad and allows for the examination of resilience across a wide range of adverse contexts. Lastly, we believe that particular features of adversity (e.g., intensity, timing, duration, controllability, and globality) shape resilience and thus need to be considered.

Third (Table 1, row 3), we focus on resilience as an outcome and are interested in which factors and processes predict resilience outcomes. In terms of which outcomes we consider, we focus on psychological health outcomes, which we characterize comprehensively along the dimensions of ill-being (e.g., depression) and well-being (e.g., life satisfaction). Thus, resilience can encompass lack of ill-being and/or presence of well-being.

In terms of applying a criterion for functioning (Table 1, row 4)—that is, where on the *y*-axis of Figure 1 one is considered to be resilient—we define resilience in relative terms, whereby a person exhibiting relatively little disruption in functioning is resilient. As depicted in the green shading of Figure 1, someone could exhibit high levels of psychological health, moderate levels of psychological health, or even some disruptions in psychological health and still be considered resilient because they exhibit less disruption than would be expected within a given cultural context for a particular adversity. This approach is based on the notion that cultural contexts differ in their understanding of what constitutes adversity and functioning (Hiebel et al. 2021). At the same time, this perspective is maximally inclusive of what is considered resilience, thus allowing us to speak to the maximum range of predictors of resilience. As indicated by the graded green and red backgrounds in Figure 1, we assume people to fall along a continuum of more versus less resilient outcomes rather than into discrete types. This expectation is driven by the observation that psychological health indicators (e.g., depression, satisfaction with life) are generally best described as continua (Kalisch et al. 2015).

Last, we consider when resilience outcomes must first appear relative to adversity onset (Table 1, row 5) and for how long they must be present (Table 1, row 6). Here we adopt a broad view, which allows us to be inclusive in our consideration of resilience. More specifically, in our view, resilience can first emerge during, immediately after, or any time after the onset of adversity. Most (but not all) people experience an initial, and at times prolonged, decrease in psychological health after experiencing adversity, and this initial decrease in health does not preclude the possibility of resilience according to our criteria. In terms of duration, we define resilience as a relatively lasting outcome (e.g., absence of lasting depression) rather than a short-term one (e.g., absence of transient depressed mood). While there is no discrete boundary defining what constitutes a lasting outcome (as indicated by the graded shading in Figure 1), an outcome should last at least 1 week to be distinguished from, for instance, a transient mood.

## PSYCHOLOGICAL APPROACHES TO EXPLAINING RESILIENCE

Now that we have reviewed the conceptual space of resilience and made explicit our location in it, we turn to the primary question that motivates this article: What psychological processes explain resilience? Two major research approaches have provided important insights into this question: the stress and coping approach and the emotion and emotion-regulation approach. We next describe each approach, highlighting their unique features, contributions, and limitations. We then present an affect-regulation framework that integrates the two approaches.

### The Stress and Coping Approach

Research on coping with stress was guided by the pioneering work of Lazarus, Folkman, and others, who emphasized the transactions between the person and the environment (Folkman 1984, Lazarus 1993). Lazarus & Folkman (1984, p. 19) defined stress as the “relationship between the person and the environment that is appraised by the person as taxing or exceeding [their] resources and endangering [their] well-being.” This highlights the idea that aspects of an individual (i.e., appraisal) interact with aspects of the environment (i.e., a stressor), and together they give rise to stress responses (changes in subjective experience, cognition, behavior, and physiology; Epel et al. 2018). When encountering stressors, people often engage in coping, “a person’s ongoing efforts in thought and action” to manage stressors and stress responses (Lazarus 1993, p. 8). From this perspective, to explain resilience we must understand what constitutes adaptive coping.

Four key features, summarized in Table 2, characterize research following the coping approach. The first feature is grounded in a stress framework, from which stems a naturalistic perspective that aims to capture the richness of people’s stressors and their responses to them. While early research involved experiments in animals in order to delineate the biological underpinnings of the stress response (for a review, see Szabo et al. 2012), contemporary research has examined a wide range of real-world stressors such as chronic illness and caregiving (e.g., Moskowitz et al. 1996), bereavement (e.g., Nolen-Hoeksema et al. 1994), and natural disasters (e.g., Holman & Silver 1998) outside the laboratory. Within this stress framework, individuals’ stress responses are typically conceived of as negative in valence and encompassing multiple response domains including experience, behavior, cognition, and physiology (Delongis et al. 1988, Lazarus & Folkman 1984, Moskowitz et al. 1996). In addition, the coping approach has often examined both psychological (e.g., affective experience) as well as biological (e.g., immune function, hormones) mechanisms of resilience outcomes (Epel et al. 1998). This approach has also examined the relationships between coping and a wide range of resilience outcomes, including psychological health (e.g., depression, well-being) and physical health (e.g., symptoms of illness; Epel et al. 1998).

The second key feature of the coping approach is its interest in comprehensively studying a wide range of real-world coping strategies. For example, one of the most commonly used measures, the Brief COPE, measures 13 different strategies (Carver 1997). In their review, Skinner and colleagues (2003) delineated 400 coping strategies that had been measured in research. Given how difficult it is to study and understand such a large number of strategies, researchers have sought to derive broader dimensions of coping (Carver & Connor-Smith 2010, Compas et al. 2017). Although there is no final consensus on broader coping dimensions, three of the most commonly used classification systems include problem-focused versus emotion-focused coping (i.e., targeting a situation versus one’s emotions; Folkman & Lazarus 1980), approach versus avoidance coping (engaging actively with versus avoiding an adverse event; Carver & Connor-Smith 2010), and cognitive versus behavioral coping (changing thoughts versus changing behaviors; Skinner et al. 2003). Of these three, the distinction between problem-focused and emotion-focused coping is the most utilized (Lazarus 1993, Skinner et al. 2003); however, research in this area often emphasizes the idea that problem- and emotion-focused coping are not mutually exclusive (Carver & Connor-Smith 2010, Folkman & Lazarus 1980, Lazarus 1999).

A third key feature of the coping approach stems from its transactional view, whereby the context within which coping occurs is explicitly considered. This emphasis leads to the idea that no particular coping strategy is inherently adaptive or maladaptive. Instead, the adaptiveness of coping strategies is shaped by the context in which they are used (Folkman & Lazarus 1980), and resilience is more likely to occur when there is greater fit between specific features of the stressors and the type of coping deployed (Cheng et al. 2014, Park et al. 2004). For example, people who use problem-focused coping in response to relatively uncontrollable adversity report higher levels of psychological symptoms compared to those who use problem-focused coping in response to relatively controllable adversity (Forsythe & Compas 1987, Park et al. 2001). In these ways, the coping approach has emphasized the importance of context and has sought to characterize and empirically study its role in shaping resilience.

Fourth, methodologically, with some early exceptions (e.g., Lazarus & Opton 1966), the methods used in coping research have typically followed its naturalistic emphasis on real-life stressors and responses to them, and they have often utilized field studies and self-report surveys to assess the naturalistic use of coping in response to stressors (Austenfeld & Stanton 2004, Lazarus 2000, Moskowitz et al. 1996). Finally, the coping approach has relied on longitudinal studies that allow for the examination of long-term relationships between coping and resilience (Austenfeld & Stanton 2004, Lazarus 2000). Taken as a whole, this naturalistic approach has produced findings with high ecological validity that provide a rich view of how coping is linked with resilience across a wide variety of adverse events, coping strategies, mechanisms, and indicators of resilience.

At the same time, the coping approach has limitations. First, within the context of the stress framework, some have noted that defining stressors via individuals’ subjective appraisals can lead to tautology of stressor and stress response (Epel et al. 2018, Lazarus et al. 1985). In addition, the stress framework typically considers stressors and stress responses as being broadly negative in valence, while neglecting discrete emotional states (Lazarus 1993, Lerner et al. 2015). Second, while the broad categories of coping described above are comprehensive, they also contain heterogeneous coping strategies that may exert different short-term effects and, in turn, differentially predict resilience (Carver et al. 1989). In other words, the categories of, for example, problem- and emotion-focused coping might be too broad. At the same time, the potential differential effects of strategies within these broader categories have not been systematically and comprehensively examined, in part because different researchers have identified different strategies as important (see Skinner et al. 2003) and have used different measures with different items and definitions, making them difficult to organize and synthesize (Austenfeld & Stanton 2004, Compas et al. 2017). Thus, comprehensiveness and breadth result in trade-offs in terms of focus and specificity. Third, although the coping approach has yielded findings high in ecological validity, the use of correlational, self-report methods to assess coping can limit the internal validity of key findings and lacks precision regarding how processes unfold in time.

### The Emotion and Emotion-Regulation Approach

The emotion-regulation approach emerged in the 1990s, in part as an outgrowth of the coping approach (Gross 1999, 2015). Although debates about the definition of emotion remain (Barrett 2006), a consensus has emerged that defines emotion as a response to any stimulus involving a valuation (e.g., good for me or bad for me) that involves loosely coupled changes in subjective experience, cognition, behavior, and peripheral physiology that unfold over a relatively short period of time (Epel et al. 2018, Gross 2015). Emotion regulation has been defined as attempts to influence which emotions people have, when they have them, and how they experience or express them (Gross 1998, 2015). While the emotion-regulation approach is broad in terms of considering emotions across different situations and stimuli, this approach is relevant to resilience in that people regulate the emotions they experience in response to adversity, which, in turn, is heavily implicated in their functioning (Aldao et al. 2010, Compas et al. 2017, Troy & Mauss 2011). From this perspective, to explain resilience we must understand what constitutes adaptive emotion regulation (e.g., Troy & Mauss 2011).

Four key features of the emotion-regulation approach are summarized in Table 2. First, its roots in an emotion framework lead this approach to consider both positively and negatively valenced emotional states (Russell & Barrett 1999, Tellegen et al. 1999); distinguish among discrete emotions such as happiness, sadness, and anxiety (Levenson 2011); and consider changes in multiple response domains that include experience, behavior, cognition, and physiology (Mauss et al. 2005). In addition, this approach has typically considered the psychological mechanisms of resilience outcomes (e.g., affective experience; Troy & Mauss 2011), and has often examined resilience outcomes in terms of psychological health (e.g., depression, anxiety; Aldao et al. 2010). The second key feature of the emotion-regulation approach is that it typically involves conceptually grounded distinctions among a relatively small number of well-defined families of emotion-regulation strategies. For example, the process model of emotion regulation distinguishes among families of emotion regulation based on which aspect of the emotion-generation process is targeted (Gross 1998, 2015). According to the process model, emotional responses arise as the result of the particular features of a situation one encounters, of how one directs one’s attention toward or away from particular stimuli, and of how one appraises stimuli. From this perspective, each of the elements involved in generating an emotional response—situation, attention, appraisals—and the emotional response itself can become targets for emotion regulation (Gross 1998, 2015). Situation change involves choosing or avoiding situations (referred to as situation selection) as well as altering situations (referred to as situation modification) (Livingstone & Isaacowitz 2015). Attentional deployment involves directing one’s attention toward or away from particular features of a situation (Sheppes et al. 2014). Cognitive change involves changing one’s interpretations or appraisals of a situation (Gross 1998, Troy & Mauss 2011). Finally, response modification involves changing one’s emotional response (including its behavioral, experiential, and physiological components) once the emotional response has begun to unfold (Gross 1998). Importantly, the process model of emotion regulation allows one to categorize emotion-regulation families and to make predictions about their short-term consequences (Gross 1998) and longer-term correlates with functioning (Aldao et al. 2010). Thus, the emotion-regulation approach has generated knowledge high in specificity and focuses on a small number of conceptually derived strategies.

A third key feature of the emotion-regulation approach emerges in contrast to the coping approach and its emphasis on transactions with the context. Instead, this approach has typically focused on understanding the main effects of particular emotion-regulation strategies on key outcomes without explicitly considering the context. In particular, this approach has studied the effects of emotion regulation with regard to a wide range of outcomes such as subjective experience, behavior, physiology, well-being, health, and relationship outcomes (Aldao et al. 2010, DeSteno et al. 2013, John & Gross 2004, Troy & Mauss 2011, Webb et al. 2012). This focus has led to an emphasis on understanding main effects of particular regulatory strategies at the individual level, such as when benefits of reappraisal in general are contrasted to the disadvantages of suppression in general, without explicitly considering context (Gross & John 2003, Richards & Gross 2000).

Fourth, with some exceptions (e.g., John & Gross 2004, Srivastava et al. 2009), emotion-regulation researchers emphasize experimental designs and laboratory approaches to examine the causal effects of particular emotion-regulation strategies (Webb et al. 2012). These investigations have included measures of self-reported experience of emotions as well as autonomic-physiological, neural, behavioral, social, and peer-rated responses, and they often examine temporally fine-grained dynamic processes (Butler et al. 2003, Gross 1998, Kalokerinos et al. 2017, Mendes et al. 2003, Sheppes et al. 2014, Troy et al. 2018). Taken together, these studies constitute a corpus of findings high in internal validity and temporal precision, which allow researchers to draw causal conclusions about the short-term effects of particular regulatory strategies on a wide range of outcomes.

The emotion-regulation approach also has limitations. First, there has been much less consideration of real-life adversity, limiting ecological validity. In addition, while many studies have examined psychological health as a key outcome, there has been much less consideration of physical health outcomes in comparison with the coping approach (cf. DeSteno et al. 2013). Second, although the process model can in theory accommodate a large number of emotion-regulation strategies, there has been a narrower empirical emphasis on reappraisal and suppression, often to the exclusion of other emotion-regulation strategies such as situation change, which limits comprehensiveness and breadth (Webb et al. 2012). Similarly, relatively fewer studies have examined spontaneously deployed (rather than instructed) emotion-regulation strategies outside of the laboratory (for recent exceptions, see Blanke et al. 2020, Grommisch et al. 2020). Third, there has historically been less emphasis on understanding transactions between emotion regulation and context, with little research explicitly considering adversity and interactions with features of adversity (cf. Aldao 2013, Bonanno & Burton 2013, Doré et al. 2016). Although recently there has been a growing emphasis on the importance of context, this recognition has appeared primarily in theorizing, with empirical work lagging behind (Aldao 2013, Sheppes 2020). Fourth, because the emotion-regulation approach has emphasized the immediate consequences of specific regulatory strategies, there have been relatively fewer investigations of the longer-term implications of emotion regulation (Gross 2015, Sheppes 2020).

### An Integrative Affect-Regulation Framework for Resilience

Although the coping and emotion-regulation approaches have been critical in generating insights into resilience, each approach has its limitations. Compounding these limitations, there has been little cross-talk between the two approaches (Compas et al. 2017, John & Eng 2014). Indeed, over two decades ago, Lazarus (1999, p. 35) wrote: “Scholars and scientists concerned with stress and coping research and theory tend not to know or cite emotion research and theory, and vice versa. This separation of fields is an absurdity.” The persistence of this problem has led to siloing that has slowed the generation, consolidation, and application of knowledge about resilience.

One path forward is to integrate these approaches and unite the two research communities by building on their shared theoretical foundations. Critically, both approaches are grounded in the idea that affect is a crucial aspect of people’s responses to adversity and a key part of what makes adversity potentially harmful. Affect has been defined as any response to an internal or external stimulus that involves valuation (e.g., “Is this good for me or bad for me?”). Affect is thus a superordinate concept that includes stress responses and emotions, as well as other phenomena such as impulses and mood (Compas et al. 2017, Epel et al. 2018, Gross 2015, Marroquin et al. 2017). Furthermore, both approaches rest on the notion that, although affective responses to adversity can intuitively appear overwhelming and unalterable, they rarely—if ever —simply happen to people. In fact, according to both approaches, people engage in efforts to change the adversity they encounter and their affective responses to it: They regulate their affect (Compas et al. 2017). In turn, affect regulation (a superordinate term that includes both coping and emotion regulation, among others) provides a key to explaining resilience: Affect regulation allows people to agentically change how they respond to adversity and the trajectory they are on, either away from or toward resilience (see Figure 1).

**Figure 2:**
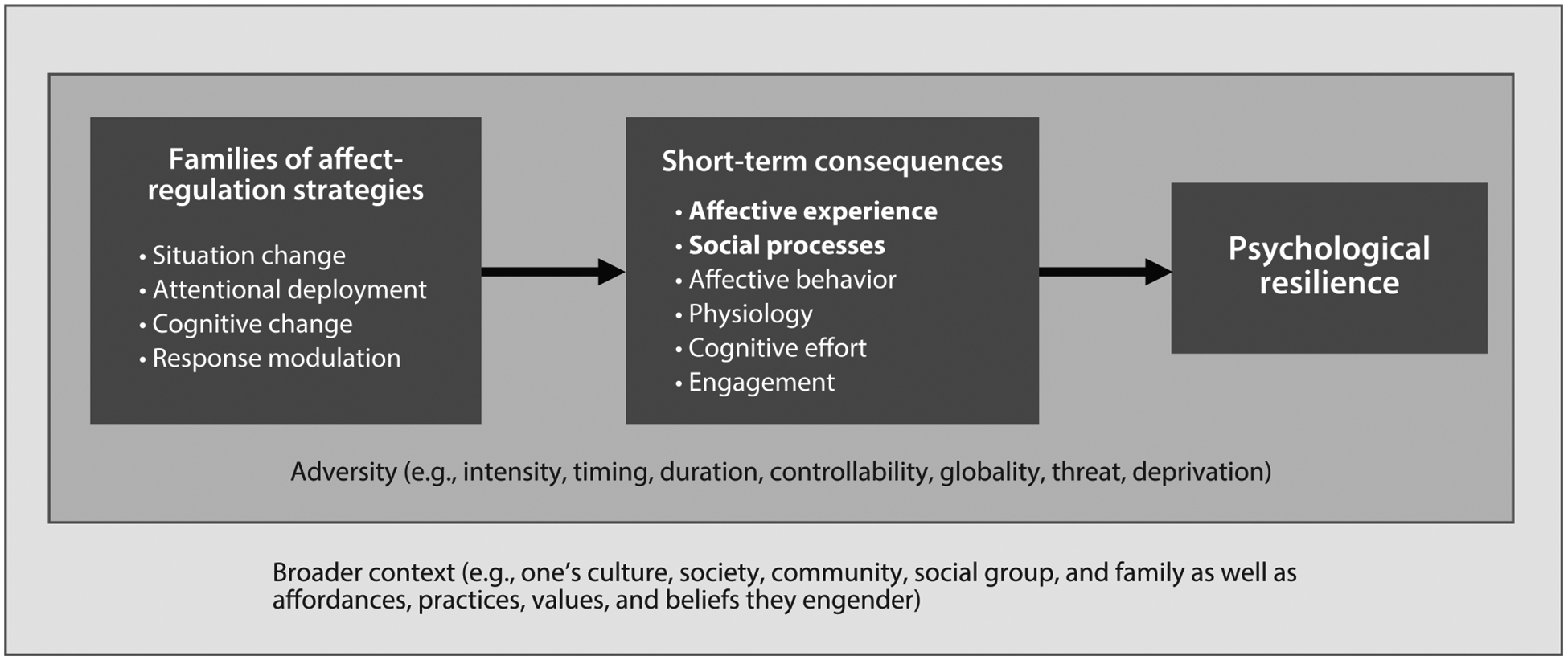
An affect-regulation framework of psychological resilience. Families of affect-regulation strategies predict short-term consequences, which predict resilience. Features of adversity influence affect regulation, its short-term consequences, and its links with resilience, as indicated by the darker-gray box. The broader context, indicated by the larger lighter-gray box, encompasses all other aspects of the framework as it influences features of adversity as well as affect regulation, its short-term consequences, its links with resilience, and even what constitutes resilience in the first place. The broader context can also give rise to three-way interactions with adversity and any other aspects of the framework depicted here. Affective experience and social processes are bolded because they are the focus of the empirical review.

The view that (*a*) both coping and emotion regulation are types of affect regulation and (*b*) affect regulation is a key to explaining resilience allows us to integrate the coping and the emotion-regulation approaches. By adopting an integrative affect-regulation framework, we can draw upon the unique strengths of both approaches. For example, we can adopt the coping approach’s strong naturalistic emphasis on understanding real-world adversity and transactions with context, while also leveraging the important contributions of the process model of emotion regulation and its emphasis on conceptually derived families of regulation. This framework organizes and consolidates existing empirical insights and guides future research that capitalizes on the strengths of both approaches, leading to increased collaboration across approaches and more comprehensive knowledge. Below, we describe three key features of an integrative affect-regulation framework, including that it (*a*) distinguishes among conceptually motivated affect-regulation strategies, (*b*) delineates profiles of short-term consequences of these strategies in multiple domains, and (*c*) predicts the longer-term implications of each strategy for resilience based on its profile of short-term consequences as well as the context in which it is used (see Figure 2).

First, drawing on the emotion-regulation approach, we distinguish among families of affect-regulation strategies based on which aspect of the affect-generation process they target (Gross 2015). Thus, we distinguish four families of affect-regulation strategies: situation change, attentional deployment, cognitive change, and response modulation. These distinctions make it possible to capture important differences in short-term consequences as well as in implications for resilience without being so nuanced as to be unmanageable.

Second, as indicated in Figure 2, drawing on both approaches, we characterize affect-regulation strategies in terms of their short-term consequences for multiple domains that, accumulating over time, have critical implications for resilience. These include the domains of affective experience (including both negative and positive affective experiences; Moskowitz et al. 1996, Rankin & Sweeny 2022, Wang et al. 2021), affective behavior (Bonanno et al. 2004, Gross 1998), autonomic physiology (McRae et al. 2010, Mendes et al. 2003), social processes (including social responsiveness, closeness, connection, liking, social support, and satisfaction with interactions; Butler et al. 2003, Lazarus & Folkman 1984, Marroquin et al. 2017), cognitive effort (the amount of effort needed to implement an affect-regulation strategy; Troy et al. 2018), and engagement (awareness of, behavioral engagement with, and learning from adversity; Nes et al. 2005). To explain resilience, we must consider more than a single type of short-term consequence. To date, much of the research on short-term consequences has focused on affective experience (Webb et al. 2012). This limits what we know about other types of short-term consequences (e.g., behavior, engagement) as well as about how profiles of multiple short-term consequences jointly shape resilience. For example, if a situation-change strategy decreases the experience of negative affect but at the same time impairs social processes, this strategy could, on the whole, decrease resilience. Thus, a critical point of the integrative framework is that it is the profile of multiple short-term consequences, rather than a single type alone, that determines resilience, with different types of short-term consequences interacting with or counteracting one another.

Another critical point is that the framework distinguishes between short-term consequences and resilience. The distinction between short-term consequences and resilience outcomes can appear somewhat blurry, as is the case when a cognitive-change strategy produces repeated episodes of positive affective experience. However, short-term consequences and resilience differ in two important ways. First, they differ in timescale. Short-term means that the consequences play out on the level of minutes to hours, as a direct consequence of an instance of affect regulation. This is distinct from resilience outcomes, which can begin to emerge at any time after the onset of adversity but must last 1 week or longer to allow us to speak of an outcome rather than a transient state (i.e., a period of stable and relatively high satisfaction with life rather than a momentary positive affect). Second, the two differ conceptually such that, as indicated next, a particular short-term consequence might lead to resilience in one context but not in another.

A third key feature of the integrative framework draws on the naturalistic perspective of the coping approach and its emphasis on the transaction between the person and the situation (see Figure 2). Based on this idea, the use of affect-regulation strategies, their short-term consequences, and the relationships between affect regulation and resilience all depend on the context. As depicted in Figure 2, context can be considered in two ways. First, more narrowly, we can consider features of adversity (as shown in the darker gray box of Figure 2). These include intensity (e.g., from low-intensity events to life-threatening trauma; Bonanno et al. 2015), timing and duration (i.e., early versus later in life, acute versus chronic; Epel et al. 2018), controllability (how much one can influence whether and how an event unfolds; Haines et al. 2016, Troy et al. 2013), globality of life domains affected by the adversity (e.g., work, family, health, finances; Epel et al. 2018), and dimensions of threat and deprivation (McLaughlin & Sheridan 2016). As depicted in Figure 2, these features of adversity can influence affect regulation, the short-term consequences of affect regulation, and the relationships between affect regulation and resilience.

Beyond features of adversity, features of the context more broadly also play a foundational role (Aldao 2013, Masten & Motti-Stefanidi 2020). The broader context can be understood as a person’s culture, society, community, social group (e.g., based on race, gender, age, or socioeconomic status), and family as well as the affordances, practices, values, and beliefs they engender (Bronfenbrenner & Ceci 1994, Markus & Kitayama 1991, Masten & Motti-Stefanidi 2020, Mesquita 2001). The broader context is relevant to resilience in that, as depicted in Figure 2 by the larger box that encompasses all other aspects of our framework, it powerfully shapes the adverse events people experience as well as affect regulation, the short-term consequences of affect regulation, and its implications for resilience, and it also determines what constitutes adversity and resilience in the first place. These effects are generated by influences of the context on affordances (e.g., are people compelled to suppress their affect?), practices (e.g., which affect-regulation strategies do people use?), values (e.g., do people value affect expression?), and beliefs (e.g., do people believe they can change their affect?) relating to affect regulation. Thus, in addition to features of adversity, the broader context is another important layer that can powerfully influence all aspects of affect regulation and its links with resilience, leading to three-way interactions in which the broader context can interact with both adversity and affect regulation to shape resilience.

In sum, the integrative affect-regulation framework considers a comprehensive range of conceptually derived affect-regulation strategies. In the integrative affect-regulation framework, the effects of affect-regulation strategies on resilience cannot be understood solely through their short-term consequences in a single domain, and they are not inherently good or bad for resilience. Instead, whether a strategy increases or decreases resilience is determined by the profile of its short-term consequences in conjunction with features of adversity and the broader context. Next, we review recent empirical findings that illustrate this framework.

## AN AFFECT-REGULATION FRAMEWORK FOR RESILIENCE: EMPIRICAL FINDINGS

In this selective review, we survey recent findings that speak to the affect-regulation framework and illustrate how the framework can be useful in organizing existing findings. We consider both correlational and experimental research from both the coping and the emotion-regulation approaches. For each family of affect-regulation strategies, we highlight one strategy that has received ample empirical attention. Although affect-regulation strategies can be used with the goal of increasing or decreasing either negative or positive affect (Gross 2015, Tamir 2016), to date, the vast majority of research has focused on decreasing negative or increasing positive affect (Webb et al. 2012). This is thus our primary focus in our review. For each strategy, we review short-term consequences for affective experiences and social processes. We focus on these two domains (rather than behavior, autonomic physiology, cognitive effort, or engagement) because they have received particular attention. After reviewing short-term consequences, we review implications for resilience. At times, when little or no evidence is available that explicitly examines the affect-regulation strategy in the context of adversity, we rely on research that examines implications for psychological health in general, making the assumption that greater psychological health implies greater resilience. We conclude each section by illustrating how features of adversity and the broader context moderate the relationship between affect regulation and resilience, selecting one example in each case.

### Situation Change

Situation change acts on the affect-eliciting situation to change its impact (Gross 2015). For example, one can attend a close friend’s birthday party (situation selection) to increase experience of positive affect or can avoid talking to one’s ex-romantic partner at the party (situation modification) to reduce experience of negative affect. The expression “situation change” maps largely onto what is referred to as problem-focused or active coping—types of coping that involve modifying the source of adversity (Carver et al. 1989, Folkman & Lazarus 1980).

#### Short-term consequences of situation change.

Past investigations and a meta-analysis have shown that situation change can be used effectively to decrease the experience of negative affect (Livingstone & Isaacowitz 2015, Thuillard & Dan-Glauser 2017, Van Bockstaele et al. 2020) and increase the experience of positive affect (Livingstone & Isaacowitz 2015, Webb et al. 2012). In terms of social processes, situation change does not appear to have consistent effects. On the one hand, situation-change strategies can enhance processes such as social support, social connectedness, and closeness (e.g., bringing a friend to a stressful work event; Chow & Glaman 2013); on the other hand, they could harm social processes (e.g., choosing not to attend a stressful work event and thus socially isolating). Thus, while in principle situation change can be very beneficial to social processes, this is not necessarily so.

#### Situation change and resilience.

Many individual studies have found that engaging in situation change is critically tied to psychological health, including after adversity (e.g., Chen et al. 2018, Heckhausen et al. 2010). However, meta-analyses have demonstrated mixed results. One meta-analysis of 42 studies found a positive association between problem solving, a key form of situation change, and psychological health (Aldao et al. 2010). However, a separate meta-analysis of a different set of 34 studies found that the association depends on the specific type of situation change, with general efforts to change the situation being associated with better psychological health and hostile or aggressive efforts to change the situation being associated with worse psychological health (Penley et al. 2002). Even within studies that used the same operationalization of situation change, meta-analytic associations were small, with some studies reporting positive associations, some finding no associations, and some finding negative associations between situation change and psychological health.

These inconsistent findings suggest that the relationship between situation change and resilience is moderated by features of the context. The controllability of one’s adverse situation has been theorized to be particularly important in this regard (Folkman 1984). After all, attempts to alter an uncontrollable situation are by definition unlikely to succeed and might lead to frustration and hopelessness. Consistent with this idea, situation change has been associated with increased resilience in relatively controllable adversity (Forsythe & Compas 1987, Vitaliano et al. 1990). In contrast, in relatively uncontrollable adversity, situation change either is not significantly associated with resilience (Park et al. 2004, Vitaliano et al. 1990) or is associated with decreased resilience (Forsythe & Compas 1987, Watanabe et al. 2002). A recent systematic review found support for controllability as a moderator of the association between situation change and resilience in 9 out of 14 studies (Finkelstein-Fox & Park 2019).

### Attentional Deployment

Attentional deployment is defined as changing the way one directs one’s attention in order to change one’s affect (Gross 2015). One particular form of attentional deployment that has received a great deal of empirical attention is distraction, which involves shifting attention away from one aspect of a situation (or thought) toward another (Gross 1998, Troy & Mauss 2011). For example, one could browse the Internet for funny videos rather than thinking about an unsettling event that just happened. The coping literature has examined distraction as part of the broader category of disengagement (versus engagement) coping (Carver & Connor-Smith 2010).

#### Short-term consequences of attentional deployment.

Laboratory studies examining the short-term effects of distraction support the idea that this strategy consistently decreases negative affective experiences and increases positive affective experiences in both clinical and nonclinical samples (Brans et al. 2013, Joormann & Stanton 2016, Webb et al. 2012). While the link between distraction and social processes has received relatively little empirical attention, work showing a negative association between experiential avoidance and social engagement suggests that distraction may also impede social processes, given that distraction can be considered a form of experiential avoidance (Gerhart et al. 2014, Kelly et al. 2019). However, there is some evidence that particular forms of distraction, such as spending time with friends, can maintain or even improve social processes (Shing et al. 2016).

#### Attentional deployment and resilience.

While distraction allows people to decrease negative and increase positive affective experiences and may (in some specific cases) maintain social processes in the short term, on average, distraction appears to be negatively associated with resilience. Repeated use of avoidance strategies such as distraction is linked to impaired psychological health, including depression (Joormann & D’Avanzato 2010, Mellick et al. 2019), perhaps because these strategies do not involve resolution of the underlying cause of adversity. Experimental studies have shown that, although distraction decreases negative affective experiences in the short term, these benefits disappear or even reverse over time (Kross & Ayduk 2008, Paul et al. 2016, Thiruchselvam et al. 2011). This suggests that distraction may provide emotional relief in the short term but decrease resilience in the longer term (Campbell-Sills & Barlow 2007, Schäfer et al. 2017).

The finding that distraction might have short-term but not longer-term benefits dovetails with findings that using distraction flexibly (rather than chronically and consistently) is associated with increased resilience (Godara et al. 2021, Wen & Yoon 2019). Both findings point to the idea that temporal aspects of adversity may shape the effects of distraction on resilience. Specifically, acute or single instances of adversity may be more suitable for distraction compared to more chronic or recurring instances of adversity. For example, when using distraction in response to one instance of a distressing stimulus, people show lower-intensity negative affective responses. However, when re-exposed to stimuli previously encountered while using distraction, people’s negative affective experiences increase (Paul et al. 2016, Thiruchselvam et al. 2011). When examining resilience outcomes more specifically, research indicates that briefly distracting and garnering positivity in the immediate aftermath of adversity may increase resilience, perhaps by allowing people to take a break from a temporally bound, intense affective response (Shing et al. 2016). On the other hand, the use of distraction in response to chronic or recurring events may decrease resilience, perhaps because it reduces vigilance and impedes problem solving (Shing et al. 2016). These ideas remain largely theoretical and require further empirical investigation.

### Cognitive Change

The most commonly studied cognitive-change strategy is reappraisal (also referred to as cognitive reappraisal, positive reappraisal, or benefit finding), which involves reframing or reconstruing an emotional situation in order to change one’s affect (Doré et al. 2016, Folkman & Moskowitz 2000, Gross 2015, Rankin & Sweeny 2022). Take, for example, one’s partner asking for a divorce, which an individual might initially appraise as an overpowering event that will devastate their own and their children’s lives. Alternatively, one might see it as painful but as something that can bring new opportunities to forge a new and healthier relationship.

#### Short-term consequences of cognitive change.

In their influential work, Lazarus and Folkman argued that it is not primarily a stressful event itself but rather one’s interpretation, or appraisal, of an event that leads to a stress response (Lazarus & Folkman 1984). From this perspective, reappraisal should allow people to successfully change or even completely transform their affective experiences. These hypotheses about reappraisal’s effects on affective experience have received substantial empirical support from both experimental and correlational studies, and meta-analyses on this topic confirm that the use of reappraisal is associated with decreased experience of negative affect and increased experience of positive affect (Augustine & Hemenover 2009, Doré et al. 2016, Rankin & Sweeny 2022, Wang et al. 2021, Webb et al. 2012).

A growing body of evidence also reveals that reappraisal has beneficial impacts on social processes. When instructed to use reappraisal during a negative affect induction in the lab, people are able to maintain social responsiveness with a conversation partner (Butler et al. 2003) and show increased forgiveness in response to a relational offense (vanOyen Witvliet et al. 2010). Furthermore, those who habitually use reappraisal more (versus less) are rated as more likeable and have closer friendships (John & Gross 2004), and the instructed use of reappraisal was associated with fewer disagreements and less relationship aggression in romantic couples during the COVID-19 pandemic (Rodriguez et al. 2021).

#### Cognitive change and resilience.

Given the pattern of short-term consequences described above, it may not be surprising that reappraisal is associated with increased resilience. For example, correlational studies have shown that reappraisal is associated with maintained or even increased psychological health following a variety of adverse events (Cavanagh et al. 2014, Moskowitz et al. 1996, Troy et al. 2010). More recent studies conducted during the COVID-19 pandemic additionally show that the use of reappraisal is associated with increased resilience, including lower levels of ill-being and higher levels of well-being (Kuhlman et al. 2021, Low et al. 2021, Smith et al. 2021).

Importantly, however, context appears to moderate the link between reappraisal and resilience. In particular, the controllability of the adversity one encounters has received attention. Multiple investigations have documented that reappraisal is associated with increased resilience in relatively uncontrollable adversity but shows no or even negative associations with resilience in response to relatively controllable adversity (Haines et al. 2016, Moskowitz et al. 1996, Troy et al. 2013). These patterns may occur because people who reappraise a controllable situation are able to decrease their experience of negative affect, which may decrease one’s motivation to take direct action to change a controllable situation (Ford & Troy 2019). Indeed, recent studies confirm that reappraisal could disrupt taking political action during an election (Ford et al. 2019) and health-related behaviors during the COVID-19 pandemic (Smith et al. 2021).

### Response Modulation

Response-modulation strategies target one’s affective responses, including affective behavior (e.g., facial, body, or vocal behavior), subjective affective experience, and physiology (Gross 2015, Webb et al. 2012). Research on response modulation has focused primarily on suppression of behavior—for example, adopting a neutral facial expression to prevent one’s boss from seeing that one is angry. Thus, we focus on this form of response modulation in our review, using the term “expressive suppression” (Gross 2015).

#### Short-term consequences of response modulation.

A meta-analysis of experimental studies reveals a large effect size of expressive suppression on expressive behavior but no consistent effect on the experience of negative affect (Webb et al. 2012). Additional correlational and experience-sampling studies with higher ecological validity indicate that expressive suppression not only is ineffective for decreasing experience of negative affect but also may reduce the experience of positive affect (Heiy & Cheavens 2014, O’Toole et al. 2014, Srivastava et al. 2009).

In terms of social processes, expressive suppression has been shown to exert negative effects on important social outcomes, perhaps because it prevents people from accurately communicating affective states or because it may be at odds with cultural values pertaining to open emotional expression. Experimental and longitudinal studies have documented a wide range of disrupted social processes resulting from expressive suppression, including decreased social responsiveness and rapport (Butler et al. 2003), lower levels of perceived authenticity (English & John 2013), and lower social support and social satisfaction (Srivastava et al. 2009).

#### Response modulation and resilience.

Given the profile of short-term consequences, one would expect the use of expressive suppression to be associated, on average, with decreased resilience, and empirical data support this prediction. For example, a meta-analysis reveals that the habitual use of expressive suppression is associated with symptoms of psychopathology, including depression, anxiety, and eating disorders (Aldao et al. 2010). Fewer studies to date have examined expressive suppression specifically following adversity. However, a handful of studies have reported positive relationships between expressive suppression and symptoms of depression and anxiety following adversity, including discrimination and trauma (Cano et al. 2020, Juang et al. 2016, Moore et al. 2008).

While it is clear that expressive suppression has negative effects on resilience in many situations, these negative effects may be attenuated, absent, or even reversed in some contexts. In particular, there is accumulating evidence that one’s culture and specific cultural values moderate the relationship between expressive suppression and resilience. For example, a meta-analysis supports that expressive suppression may have a detrimental effect on resilience in individuals with Western cultural values but no effect on resilience in individuals with Eastern cultural values (Hu et al. 2014). Similarly, a prospective study in a sample of Mexican-origin adolescents found the suppression of negative emotion (but not positive emotion) predicted decreases in anhedonia symptoms of depression (Young et al. 2022). One explanation for these findings is that collectivistic-oriented cultures (e.g., Asian and Mexican), compared with more individualistic-oriented cultures (e.g., European), place a higher value on expressive suppression, especially of negative emotion, perhaps to support interpersonal harmony and social goals (Markus & Kitayama 1991, Mesquita 2001, Wilms et al. 2020).

## DIRECTIONS FOR FUTURE RESEARCH ON RESILIENCE

In closing, we discuss key directions for future research on resilience indicated by the integrative affect-regulation framework (see Figure 2). We organize these directions by discussing each of the elements of the framework, namely: affect regulation processes, their short-term consequences, the moderating role of context, and implications for resilience.

### Affect-Regulation Processes

It is important to deepen as well as broaden our understanding of affect regulation. Within families of affect regulation, emerging research and theorizing suggest potential additional distinctions that could have crucial implications for resilience. For example, several distinctions within the category of reappraisal have been proposed, including (*a*) reconstrual (changing how a situation is interpreted) versus repurposing (changing the goals to which one compares the situation) (Uusberg et al. 2019), (*b*) reappraisal aimed at increasing positive affect versus reappraisal aimed at decreasing negative affect (Folkman & Moskowitz 2000, McRae & Mauss 2016), and (*c*) reappraisal of the situation versus reappraisal of one’s emotional response to it (Jamieson et al. 2018).

It is also important to make distinctions among the processes involved in affect regulation, including identifying the need for affect regulation, selecting an affect-regulation strategy, implementing it, and monitoring its implementation (Gross 2015). Each of these processes—identification, selection, implementation success, and monitoring—likely has unique implications for resilience, and empirical work is just beginning to examine and delineate these processes (Ford et al. 2017, Sheppes et al. 2014).

There is also a need to broaden the scope of analysis. For instance, more research is needed that focuses on strategies beyond reappraisal and suppression to capture the full range of affect-regulation strategies. In addition, we need to study more complex affect-regulation strategies that do not fit neatly into one affect-regulation family. For example, emotional acceptance, which has been linked to resilience (Ford et al. 2018a), includes features of attentional deployment (e.g., a focus on the present moment), cognitive change (e.g., reframing one’s affective states in a non-judgmental way), and response modulation (e.g., fully experiencing one’s affective states without attempting to change them) (Ford et al. 2018a, Troy et al. 2018).

Finally, it will be critical to better understand the antecedents of affect regulation, including antecedents that are biological (e.g., genes; Southwick & Charney 2012), situational (e.g., environmental affordances; Suri et al. 2018, Uusberg et al. 2019), and psychological (e.g., beliefs about emotions, personality, self-efficacy, or goals; Bonanno et al. 2015, Carver & Connor-Smith 2010, John & Eng 2014). The beliefs people hold about their emotions might be especially impactful in that they simultaneously affect multiple aspects of affect regulation (Ford & Gross 2019, Zerwas et al. 2022). For example, the belief that one can control one’s emotions may shape affect regulation identification, selection, and implementation success (Ford et al. 2018b, Gutentag et al. 2017). In turn, such antecedents might be promising targets for intervention.

### Short-Term Consequences of Affect Regulation

At the level of short-term consequences, more work is needed to comprehensively document the full range of short-term consequences listed in Figure 2 and their implications for resilience. The largest body of research to date has examined the consequences of affect regulation on the experience of affect, often focusing exclusively on negative affect. Yet, examining consequences for positive affect such as joy or hope—independently of negative affect—might be an especially fruitful path to understanding resilience (Duker et al. 2022, Folkman & Moskowitz 2000, Monroy et al. 2021, Tugade & Fredrickson 2004). Beyond negative and positive affect, it will be useful to examine the consequences of affect regulation for discrete emotions. For instance, anger and sadness (Barlow et al. 2019, Shu et al. 2021) or excitement and contentment (Chim et al. 2018, Hamm et al. 2021) may have distinct implications for resilience. Furthermore, consequences other than affective experience are important in explaining resilience; yet there are still critical gaps in knowledge when it comes to behavior (especially beyond facial expressions), physiology, social processes (e.g., social support, social connectedness), cognitive effort, and engagement (e.g., awareness of, behavioral engagement with, and learning from adversity). Lastly, research is needed that concurrently examines multiple short-term consequences (e.g., affective experience, social processes, and engagement) in order to clarify how profiles of short-term consequences shape resilience.

A second aspect of a deeper understanding of the short-term consequences of affect regulation involves investigating their time course and dynamics. To date, research has heavily focused on mean-level consequences of affect regulation (e.g., how does someone feel after using reappraisal relative to after using suppression?). Yet more complex dynamics of these consequences might play a role in shaping resilience, including variability over time, trajectories over time, and relationships among multiple response systems (Willroth et al. 2020; see Dejonckheere et al. 2019 for discussion). For example, regardless of how someone feels when they first encounter adversity, the rate at which their feelings recover and how they feel over the longer term might play an important role in resilience (Leger et al. 2018).

### The Context

In our empirical review, we highlighted some of the growing number of studies that have examined the role that context plays in affect regulation and resilience. Yet, much remains to be learned. First, it will be important to more systematically and comprehensively examine features of adversity, including intensity, timing, duration, controllability, globality, and type of adversity, among others. Emerging research on reappraisal highlights the utility of this approach.

For example, some forms of reappraisal in response to discrimination have been shown to be associated with null effects on short-term affective experiences (Duker et al. 2022) and decreased psychological health (Perez & Soto 2011), perhaps because they invalidate one’s experiences or undermine one’s values (Ford & Troy 2019). To gain a more comprehensive understanding of the role of adversity and its particular features, we need more studies that consider and compare presence versus absence of adversity, levels of severity of adversity, and other features of adversity (e.g., Seery et al. 2010). In addition, we need to more deeply and comprehensively consider features of the broader context, including of people’s culture, society, community, social group membership, and family. Existing evidence illustrates that the broader context—through the affordances, practices, values, and beliefs it engenders—can powerfully shape affect regulation and its links with resilience. There are also likely to be important interactions between the broader context, adversity, and affect regulation in shaping resilience. Yet, empirical research in this area is in its infancy.

Considering context will shift how we conceptualize affect regulation’s role in resilience, moving it away from thinking of affect-regulation strategies as either inherently conducive to or harmful for resilience. Rather, we need to think of affect-regulation strategies as conducive to resilience depending on how flexibly and context-appropriately they are deployed (Bonanno & Burton 2013, Cheng et al. 2014, Ford & Troy 2019). Several recent developments have begun to capture these aspects of affect regulation, including by considering affect-regulation repertoires (Grommisch et al. 2020), affect-regulation flexibility (Birk & Bonanno 2016, Sheppes et al. 2014), within-person variability in affect-regulation use (Aldao et al. 2015, Blanke et al. 2020), and fit between affect regulation and context (e.g., Cheng et al. 2014). Considering context also highlights the critical idea that interventions can target both affect regulation and the context as ways to increase resilience. Within the affect-regulation framework, it is especially promising to build on these developments.

### Resilience Outcomes

At the level of resilience, it will be important to better understand the degree to which affect regulation has general versus specific effects on facets of resilience. One key question is illustrated by Figure 1. We can ask whether the same processes that help people avoid doing worse than expected (e.g., avoid worse ill-being; see red line in Figure 1) also help people do better than expected (e.g., gain well-being and growth; see green line in Figure 1) (Ryff & Singer 1998, Seligman 2008). Further distinctions that are important to explore involve different domains of psychological health; for example, we need to examine whether anxiety versus depression or hedonic versus eudaimonic well-being are predicted in similar or distinct ways. Lastly, resilience does not just concern psychological functioning but also physical functioning, and a major direction for research is to apply the present framework to physical health (DeSteno et al. 2013, McEwen & Wingfield 2003). Initial research indicates that likely there is at least some specificity, such that affect regulation that is helpful in one domain might not be helpful, or may even be harmful, in other domains (Miller et al. 2015).

### Concluding Comment

Human life comes with unavoidable adversity. How we deal with this adversity depends crucially on affect regulation. We offer an integrative affect-regulation framework for resilience, which builds on and integrates the coping and emotion-regulation approaches. This integrative framework broadens and deepens how we understand affect regulation, its consequences, and its embeddedness in context. Ultimately, the affect-regulation framework has the promise to guide the development and improvement of prevention and intervention efforts by suggesting precisely which aspects of affect regulation might be of use and in what contexts. This consideration of context also means that, in addition to helping with individual affect regulation, interventions should target how people relate to their context as well as provide educational, occupational, financial, and structural resources to ultimately enhance resilience at the individual, family, and community levels. Our hope is that the affect-regulation framework provides a research agenda for researchers to gain a better understanding of resilience and how we can enhance it.

## Figures and Tables

**Table 1:** The conceptual space of resilience

Conceptual question	Possible answers	Our position
1. What is the level of analysis?	Individual Group Community	We focus on the individual level.
2. How is adversity considered?	Adversity is not explicitly considered (e.g., trait measures of resilience that are used in the presence or absence of adversity). Adversity is explicitly considered: Some type of adversity must be experienced to observe resilience. Adversity can be defined objectively (e.g., checklist measures) or subjectively (e.g., individual appraisal of stress). Particular features of adversity are considered. Intensity, ranging from low (e.g., daily hassles or transient events) to high (e.g., a natural disaster or major illness) Timing (e.g., occurring early versus later in life) and duration (e.g., acute events lasting minutes to days versus chronic events lasting months to years) Controllability (e.g., how much one can influence whether and how an event occurs and unfolds over time) Additional important features: globality of life domains concerned, threat, deprivation Specific types of adverse circumstances or events are considered (e.g., job loss, divorce, natural disaster).	We consider resilience in the context of adversity. We consider features of adversity.
3. What is the nature of resilience?	Factors (e.g., relatively stable ability to adapt to adversity) Processes (e.g., deploying resources to adapt to adversity) Outcomes Individual level (e.g., greater well-being; greater psychological health; lower levels of psychopathology; fewer symptoms; absence of diagnosis; greater academic, occupational, or social achievements; reaching developmental milestones) Group/community level (e.g., greater social and economic infrastructure, greater educational and human development resources)	We distinguish factors, processes, and outcomes. Here, we define resilience as an outcome. We focus on psychological health as a resilience outcome.
4. What criterion for functioning is applied to determine resilience?	Absolute point (e.g., meets a threshold such as absence of clinical depression) Relative to other people experiencing similar adversity (e.g., less depression relative to other bereaved spouses) other people who did not experience adversity (e.g., similar functioning between an individual experiencing bereavement and an individual not experiencing bereavement) oneself (e.g., returns to or exceeds one’s pre-bereavement levels of depression) expectation in a particular cultural context and given particular adversity (e.g., less depression than one’s culture would expect given bereavement) Discrete types (someone is or is not resilient) or on a continuum (someone is more or less resilient)	We consider resilience in relation to expectations in a particular cultural context and given particular adversity. We consider resilience on a continuum (more versus less) rather than as discrete types.
5. What criterion for trajectory is applied to determine resilience (i.e., when, in relation to adversity onset, does resilience need to be present)?	At any point in time (e.g., relatively high psychological health throughout life) Beginning during adversity (e.g., relatively high psychological health during a major illness) Beginning seconds or minutes after adversity (e.g., relatively high psychological health immediately after learning about a major illness) Beginning weeks or months after adversity (e.g., relatively high psychological health 2 months after sudden job loss) Beginning years after adversity (e.g., relatively high psychological health in adulthood after abuse in childhood)	We determine resilience during, immediately after, or up to decades after adversity onset.
6. What criterion for duration is applied to determine resilience (i.e., for how long does resilience need to be present)?	Duration not considered At least seconds to minutes (e.g., positive mood lasting for several minutes) At least days to months (e.g., 2 months of relatively high psychological health) At least years to decades (e.g., several years of relatively high psychological health)	We consider durations of at least 1 week.

Six questions one encounters in thinking about resilience (first column), the range of possible answers (second column), and our position in the space (third column).

**Table 2:** Key theoretical and methodological features of the stress and coping approach and the emotion and emotion-regulation approach

Stress and coping approach	Emotion and emotion-regulation approach
1. Grounded in stress framework Consideration of wide range of real-world stressors Focus on negatively valenced stress responses Consideration of multiple domains of stress responses (e.g., subjective experience, behavior, cognition, physiology), and of both biological (e.g., viral load, immune function, hormones) and psychological (e.g., affective experience) mechanisms Consideration of psychological and physical health outcomes	1. Grounded in emotion framework Consideration of emotion-eliciting events, broadly (positive or negative) Consideration of discrete negative and positive emotions (e.g., sadness, anger, anxiety, joy, calm) Consideration of multiple domains of emotional responding (e.g., subjective experience, behavior, cognition, physiology) and of psychological (e.g., affective experience) mechanisms Consideration of psychological health outcomes, less emphasis on physical health outcomes
2. Emphasis on a wide range of real-world coping strategies (e.g., 13 strategies of the Brief COPE) or broad categories of coping strategies (e.g., emotion-focused versus problem-focused) More comprehensiveness and breadth Less focus, homogeneity, and specificity	2. Emphasis on a relatively small number of families of theoretically grounded emotion-regulation strategies (e.g., 4 families of strategies derived from the process model) Less comprehensiveness and breadth More focus, homogeneity, and specificity
3. Transactional approach Greater consideration of context (e.g., controllability of stressors)	3. Process-based approach Emphasis on main effects of emotion regulation, less consideration of context
4. Naturalistic emphasis Greater reliance on surveys and correlational methods to assess coping Greater emphasis on longer-term relationships between coping and resilience Greater ecological and lower internal validity	4. Emphasis on controlled laboratory contexts Greater reliance on laboratory studies with experimental manipulations of emotion regulation Greater emphasis on shorter-term causal effects of emotion regulation and examinations of temporally fine-grained dynamic processes Greater internal and lower ecological validity

The table indicates differences in emphasis and central tendency between the two approaches, not categorical differences.

## SUMMARY POINTS

1. Research on the processes that explain psychological resilience has come from two major sources: the stress and coping approach and the emotion and emotion-regulation approach. Each approach has yielded important insights but is limited by (*a*) a lack of conceptual clarity and (*b*) a lack of integration of the two approaches.
2. To contribute to conceptual clarity, we review six questions encountered in research on resilience and provide an overview of the conceptual space in which these questions can be answered. We then locate our own approach within this conceptual space.
3. To contribute to integration, we propose an affect-regulation framework that joins insights from the two approaches.
4. We use this integrative framework to structure our empirical review of findings on coping, emotion regulation, and resilience. This review (*a*) focuses on four theoretically derived families of affect-regulation strategies, (*b*) reviews the short-term consequences of these strategies for affective experience and social processes, (*c*) reviews the relationships between affect-regulation strategies and resilience, and (*d*) highlights how links between affect regulation and resilience are powerfully shaped by aspects of adversity and of the broader context.
5. A key insight from the integrative affect-regulation framework is that the effects of affect-regulation strategies on resilience cannot be understood solely through their short-term consequences in a single domain, and they are not inherently good or bad for resilience. Instead, whether a strategy increases or decreases resilience is determined by the profile of its short-term consequences in conjunction with features of adversity (e.g., intensity, controllability of an adverse event) and of the broader context (e.g., one’s culture, social group, family).
6. The integrative affect-regulation framework capitalizes on the complementary strengths and unique contributions of the coping and emotion-regulation approaches, organizes existing empirical insights, and guides future research on resilience by providing an agenda for researchers to gain a better understanding of resilience and how we can enhance it.

## FUTURE ISSUES

1. More work is needed that examines resilience at the group and community levels, in addition to the individual level we focused on here.
2. Researchers should examine a wider range of affect-regulation strategies (e.g., situation selection, acceptance), a wider range of processes (e.g., affect-regulation identification, selection, implementation, and monitoring processes), and antecedents of affect regulation (e.g., beliefs).
3. More work is needed that documents the full range of short-term consequences of affect regulation that are relevant for resilience (e.g., behavior, social processes, engagement) and that explores more complex dynamics within these consequences (e.g., variability and trajectories over time).
4. We need to conduct comprehensive investigations of the role of context, including features of adversity and of the broader context, and how it shapes affect regulation, its short-term consequences, and its links with resilience.
5. Future research should examine multiple resilience outcomes (e.g., well-being, mental health, physical health) and how they relate to one another.
6. A key priority moving forward is to build a comprehensive, causal, and mechanistic understanding of affect regulation and resilience by employing longitudinal or intervention studies that measure or manipulate affect regulation, examine the mechanistic role of short-term consequences, carefully consider theoretically motivated aspects of the context, and document longer-term effects on resilience.
7. Existing and emerging findings produced within the affect-regulation framework can be used to increase resilience by developing and improving prevention and intervention programs that target affect regulation and its context.

## Abbreviations

Adversity: circumstance or event that in a given cultural context would be expected to tax or exceed a person’s resources and disrupt functioning
Psychological resilience: faring better than would be expected in terms of psychological functioning in a given cultural context following adversity
Stressor: an event that is appraised as taxing or exceeding resources and endangering well-being
Stress response: a response to a stressor, encompassing changes in subjective experience, cognition, behavior, and physiology; typically negative in valence and unfolding over a short time
Coping: behavioral and cognitive strategies used to manage stressors or stress responses
Emotion: a response to an internal or external stimulus involving a valuation and encompassing changes in subjective experience, cognition, behavior, and physiology
Emotion regulation: strategies used to alter one’s emotions, including attempts to change subjective experience, cognition, behavior, physiology, or the environment
Affect: any response to an internal or external stimulus involving a valuation; stress and emotion can both be considered subsets of affect
Affect regulation: strategies used to alter affect, including attempts to change subjective experience, cognition, behavior, physiology, or the environment; coping and emotion regulation are subsets of emotion regulation
